# Continuous barbed suturing improves early recovery after primary total knee arthroplasty: a randomised controlled trial

**DOI:** 10.1186/s13018-025-06471-y

**Published:** 2026-01-25

**Authors:** Juan Miguel Gómez-Palomo, Irene Montañez-Marín, Amparo Zamora-Mogollo, Carmen Tara-Abad, Silvia Sofía Irizar-Jiménez, Ana Martínez-Crespo

**Affiliations:** 1 Department of Orthopedic Surgery and Traumatology, Virgen de la Victoria University Hospital, Campus Teatinos, S/N, 29010 Málaga, Spain; 2 Biomedical Research Institute of Málaga (IBIMA), Severo Ochoa Avenue, 35, 29590 Málaga, Spain; 3 Faculty of Health Sciences, University of Málaga, Campus de Teatinos, Arquitecto Francisco Peñalosa 3, 29071 Málaga, Spain

**Keywords:** Total knee arthroplasty, Wound closure, Barbed sutures, Postoperative pain, Functional outcome, Randomised controlled trial.

## Abstract

**Purpose:**

To evaluate whether continuous barbed suturing improves postoperative pain, closure efficiency, and early functional outcomes compared to conventional interrupted suturing in primary total knee arthroplasty (TKA).

**Methods:**

In this double-blinded randomised controlled trial, 143 patients undergoing primary TKA were assigned to continuous barbed (*n* = 72) or interrupted absorbable (*n* = 71) sutures for arthrotomy and subcutaneous closure. The primary endpoints were closure time and postoperative pain at 24 h; superiority testing was pre-specified only for VAS at 24 h (one-sided t-test, α = 0.025), whereas closure time was analysed two-sided (α = 0.05). Secondary outcomes included wound healing time, 6-month functional gain (Hospital for Special Surgery score), quality of life (EQ-5D), satisfaction, and complications.

**Results:**

Continuous suturing significantly reduced closure times for both arthrotomy (4.2 ± 1.6 vs 6.5 ± 7.7 min; *p* < 0.001) and subcutaneous layers (4.8 ± 1.9 vs 5.6 ± 1.4 min; *p* < 0.001). Pain at 24 h was significantly lower in the continuous group (VAS 2.9 ± 2.1 vs 4.0 ± 2.1; *p* = 0.017). The continuous group also showed faster wound healing (22.3 vs 24.8 days; *p* = 0.012) and greater 6-month HSS improvement (24.5 vs 16.0 points; *p* = 0.040). No significant differences were observed in complication rates, satisfaction, or quality of life.

**Conclusion:**

Continuous barbed suturing improves surgical efficiency, reduces early postoperative pain, accelerates wound healing, and enhances functional recovery without increasing complication rates. This is the first randomised trial to demonstrate superior functional recovery (HSS score) with barbed continuous closure over traditional interrupted techniques, supporting its broader adoption in primary TKA.

**Level of evidence:**

Level I, randomised controlled trial.

## Introduction

Total knee arthroplasty (TKA) is a widely performed and cost-effective procedure for advanced osteoarthritis, aiming to relieve pain, restore joint function, and improve quality of life [1]. Despite progress in implant design and surgical technique, wound-related complications such as infection, delayed healing, and dehiscence remain relevant concerns, particularly during the early postoperative phase [2].

Among perioperative variables influencing recovery, the method of wound closure has gained increasing attention. Continuous sutures offer theoretical advantages such as reduced closure time and more uniform tension distribution, while interrupted sutures may allow better control of local tension and drainage [3]. However, evidence from well-designed randomised controlled trials (RCTs) comparing these techniques in TKA remains limited [4], and their potential impact on operative efficiency and early patient-reported outcomes has not been conclusively established.

Expert consensus and surgical guidelines have recommended continuous barbed sutures based on perceived benefits in closure speed, reduced complications, and ergonomic advantages [5–8]. Nevertheless, most of these recommendations rely on expert opinion or observational data, with only a few clinical trials directly addressing objective outcomes such as pain, efficiency, or recovery.

In this context, our study aimed to provide high-level evidence through a prospective, randomised trial comparing continuous versus interrupted sutures in primary TKA, using two pre-specified co-primary endpoints: closure time and early postoperative pain (VAS at 24 h). While both endpoints were considered clinically meaningful, the sample size was specifically powered to detect differences in pain.

We hypothesised that continuous suturing would significantly reduce wound closure time and postoperative pain without increasing complications, thereby supporting its broader adoption in routine clinical practice.

## Materials and methods

### Study design and ethical approval

This was a single-centre, prospective, double-blind, randomised controlled trial (RCT) (patients and outcome assessors were blinded) conducted at Virgen de la Victoria University Hospital (Málaga, Spain) between 1 January and 31 December 2024. The objective was to compare operative efficiency and early postoperative outcomes of continuous barbed versus interrupted absorbable suturing for arthrotomy and subcutaneous closure during primary total knee arthroplasty (TKA).

The study was approved by the Provincial Research Ethics Committee of Málaga (approval code: SICEIA-2024-001425) and conducted in accordance with the Declaration of Helsinki, Good Clinical Practice guidelines, and national data protection laws (Ley Orgánica 3/2018) and the EU General Data Protection Regulation (Regulation (EU) 2016/679). All participants provided written informed consent before enrolment. The trial is reported in accordance with CONSORT guidelines.

### Eligibility criteria

Eligible participants were adults aged 50–80 years with symptomatic knee osteoarthritis (Kellgren–Lawrence grade III–IV) and a formal indication for unilateral primary TKA. Additional inclusion criteria included a body mass index (BMI) ≤ 35 kg/m², no prior surgery on the affected knee, ASA physical status I–III, and the ability to complete follow-up for at least 6 months.

Exclusion criteria included previous arthroplasty or open knee surgery on the same limb, current infection or immunosuppressive therapy (e.g. HIV, chemotherapy), severe uncontrolled diabetes or peripheral vascular disease, active inflammatory arthropathy, chronic corticosteroid use, non-reversible anticoagulation, or any intraoperative deviation from the closure protocol. Patients unable to provide informed consent or to comply with the follow-up schedule were also excluded.

### Randomisation and blinding

Randomisation was performed in a 1:1 ratio using a computer-generated block scheme (block size = 10), managed by an independent statistician. Group allocation was concealed in sequentially numbered opaque envelopes, opened intraoperatively by the circulating nurse after implant placement.

The study was double-blinded: patients and outcome assessors were blinded to group allocation. Blinding of surgeons was not feasible due to the nature of the intervention.

### Surgical procedure

All procedures were performed by two senior orthopaedic surgeons with over 10 years of experience in knee arthroplasty. A standard medial parapatellar approach was used in all cases under spinal anaesthesia and pneumatic tourniquet (300 mmHg). A single cemented implant model was used for all patients.

Prophylaxis included intravenous tranexamic acid (1 g before incision and repeated at 3 h), intravenous cefazolin, and thromboprophylaxis with subcutaneous enoxaparin according to institutional protocol.

### Closure techniques

The only interventional difference between groups was the suturing method used for arthrotomy and subcutaneous closure:

- *Continuous group* (*n* = 72): A running suture technique using a barbed monofilament absorbable suture (Stratafix® PDS Plus Symmetric 0, Ethicon) was applied. The suture was anchored proximally and closed distally with a locking loop, maintaining uniform tension throughout the wound.
- *Interrupted group* (*n* = 71): Closure was achieved using a braided absorbable suture (Vicryl® 0, Ethicon), applied in individual stitches spaced approximately 1 cm apart, each secured with a separate knot.

In both groups, skin closure was performed using staples (3 M™ Precise™), removed on postoperative day 14. No topical adhesives were applied.

### Postoperative care and rehabilitation

All patients received standardised analgesia (paracetamol and NSAIDs, with opioid rescue as needed) and a uniform physiotherapy protocol, including mobilisation and full weight-bearing from postoperative day 1. Closure of deep layers was routinely performed with the knee in 30° flexion. Wound inspection followed institutional protocols.

### Outcome measures

Clinical assessments were performed by trained assessors blinded to group allocation at baseline and postoperative days 7, 14, and 30, and months 3 and 6 using standardised case-report forms.

- Primary outcomes
  - Closure time, recorded in minutes with a stopwatch from the start to the end of each layer (arthrotomy and subcutaneous).
  - Postoperative pain, measured using the Visual Analogue Scale (VAS) at 24 h.
  - Both outcomes were pre-specified as co-primary endpoints; however, sample-size calculation and hypothesis testing were based solely on the VAS pain score.
- Secondary outcomes
  - Time to complete wound healing, defined as full epithelialisation without crusting, discharge, erythema, or wound-edge separation. Assessment was performed by direct inspection at scheduled outpatient follow-ups; visit timing was recorded and considered in the interpretation.
  - Knee function at 6 months, assessed using the Hospital for Special Surgery (HSS) score.
  - Knee range of motion (ROM), defined as active knee flexion in degrees at 6 months and obtained from physiotherapy records, analysed as an exploratory endpoint.
  - Health-related quality of life (HRQoL) via EQ-5D-3 L index at 6 months.
  - Patient satisfaction at 3 months, measured on a 3-point Likert scale.
  - Wound-related complications, including superficial/deep infection (CDC criteria), dehiscence, or reoperation related to the wound.

Hospital resource use and costs were not prospectively collected, and no formal economic evaluation was pre-specified; economic implications are considered qualitatively in the Discussion.

### Sample size calculation

The study was powered to detect superiority of the continuous suture technique over the interrupted method in terms of VAS pain score at 24 h. A sample size of 64 patients per group was required to detect a minimum clinically relevant difference of 1.0 point (standard deviation = 2.0), assuming a one-sided two-sample t-test with a significance level (α) of 0.025 and power (1–β) of 80%. Accounting for an estimated 10% attrition rate, 143 patients were recruited.

### Statistical analysis

Statistical analyses were performed using IBM SPSS Statistics v25.0 (IBM Corp., Armonk, NY, USA). Normality was assessed using the Shapiro–Wilk test. Continuous variables were reported as mean ± standard deviation (SD) or median [interquartile range], and categorical variables as frequencies and percentages.

Superiority testing for the primary outcome (VAS pain score at 24 h) was conducted using a one-sided two-sample t-test with a significance level of 0.025. The null hypothesis (H₀) stated that the mean VAS score in the continuous group was greater than or equal to that in the interrupted group (µ_continuous – µ_interrupted ≥ 0), while the alternative hypothesis (H₁) posited that the continuous group would report lower pain scores (µ_continuous – µ_interrupted < 0).

The co-primary outcome of closure time was analysed using a two-sided t-test with α = 0.05, with distributional assumptions checked; the Mann–Whitney U test was used if assumptions were violated. Other secondary outcomes were compared using Student’s t-test, Mann–Whitney U, Chi-square or Fisher’s exact test, as appropriate. Knee range of motion (ROM) at 6 months was analysed as an exploratory endpoint using the Mann–Whitney U test and summarised as median [IQR] and mean ± SD. A p-value < 0.05 (two-sided unless otherwise specified) was considered statistically significant for these comparisons.

## Results

**Fig. 1:**
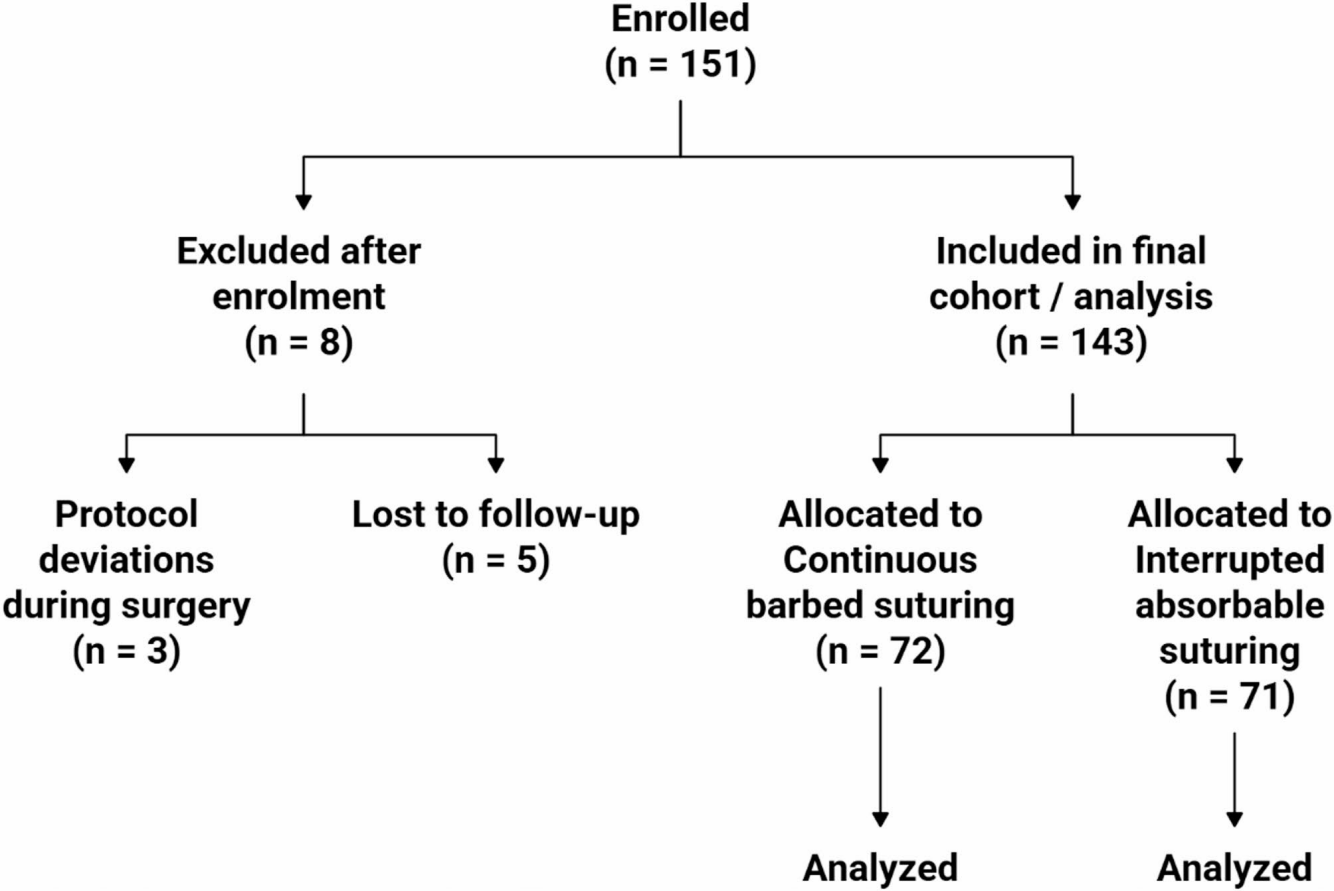
CONSORT flow diagram. Trial profile illustrating participant flow from enrolment to analysis

A total of 151 patients were initially enrolled. Eight patients (5.3%) were excluded due to loss to follow-up (*n* = 5) or protocol deviations during surgery (*n* = 3). The final cohort consisted of 143 patients: 72 in the continuous suture group and 71 in the interrupted suture group. The mean duration of follow-up was 7.0 ± 2.2 months. An expanded CONSORT flow diagram has been added to illustrate patient progress from enrolment to analysis (Fig. 1).

### Baseline characteristics

Baseline demographic and clinical characteristics were comparable between groups (Table 1). No significant differences were observed in age (68.8 ± 10.4 vs 70.4 ± 6.7 years, *p* = 0.560), sex distribution (44.4% vs 46.5% female, *p* = 0.784), BMI (29.2 ± 4.5 vs 29.6 ± 4.5 kg/m², *p* = 0.600), smoking status (13.2% vs 11.4%, *p* = 0.784), or Charlson comorbidity index (3.6 ± 1.5 vs 3.6 ± 1.2, *p* = 0.939).

**Table 1:** Baseline demographic and clinical characteristics of the study population

Variable	Continuous (*n* = 72)	Interrupted (*n* = 71)	*p*-value
Age (years)	68.8 ± 10.4	70.4 ± 6.7	0.560
Female sex (%)	44.4	46.5	0.784
Body mass index (kg/m²)	29.2 ± 4.5	29.6 ± 4.5	0.600
Current smoker (%)	13.2	11.4	0.784
Charlson comorbidity index	3.6 ± 1.5	3.6 ± 1.2	0.939

Values are presented as mean ± standard deviation or percentages. *p*-values were calculated using the Mann–Whitney U test (continuous variables) and the Chi-square test (categorical variables). No significant differences were observed between groups

### Primary outcomes

Closure time was significantly reduced in the continuous suture group compared to the interrupted group. Mean arthrotomy closure time was 4.2 ± 1.6 min vs 6.5 ± 7.7 min (two-sided *p* < 0.001), and subcutaneous closure was also faster (4.8 ± 1.9 vs 5.6 ± 1.4 min, two-sided *p* < 0.001) (Table 2).

**Table 2:** Surgical closure times

Closure layer	Continuous (min)	Interrupted (min)	*p*-value
Arthrotomy	4.2 ± 1.6	6.5 ± 7.7	< 0.001
Subcutaneous tissue	4.8 ± 1.9	5.6 ± 1.4	< 0.001

Closure times were measured intraoperatively using a stopwatch. Values are expressed as mean ± standard deviation. Group comparisons were conducted using the Mann–Whitney U test due to the presence of outliers and non-normal distribution in the interrupted group

**Fig. 2:**
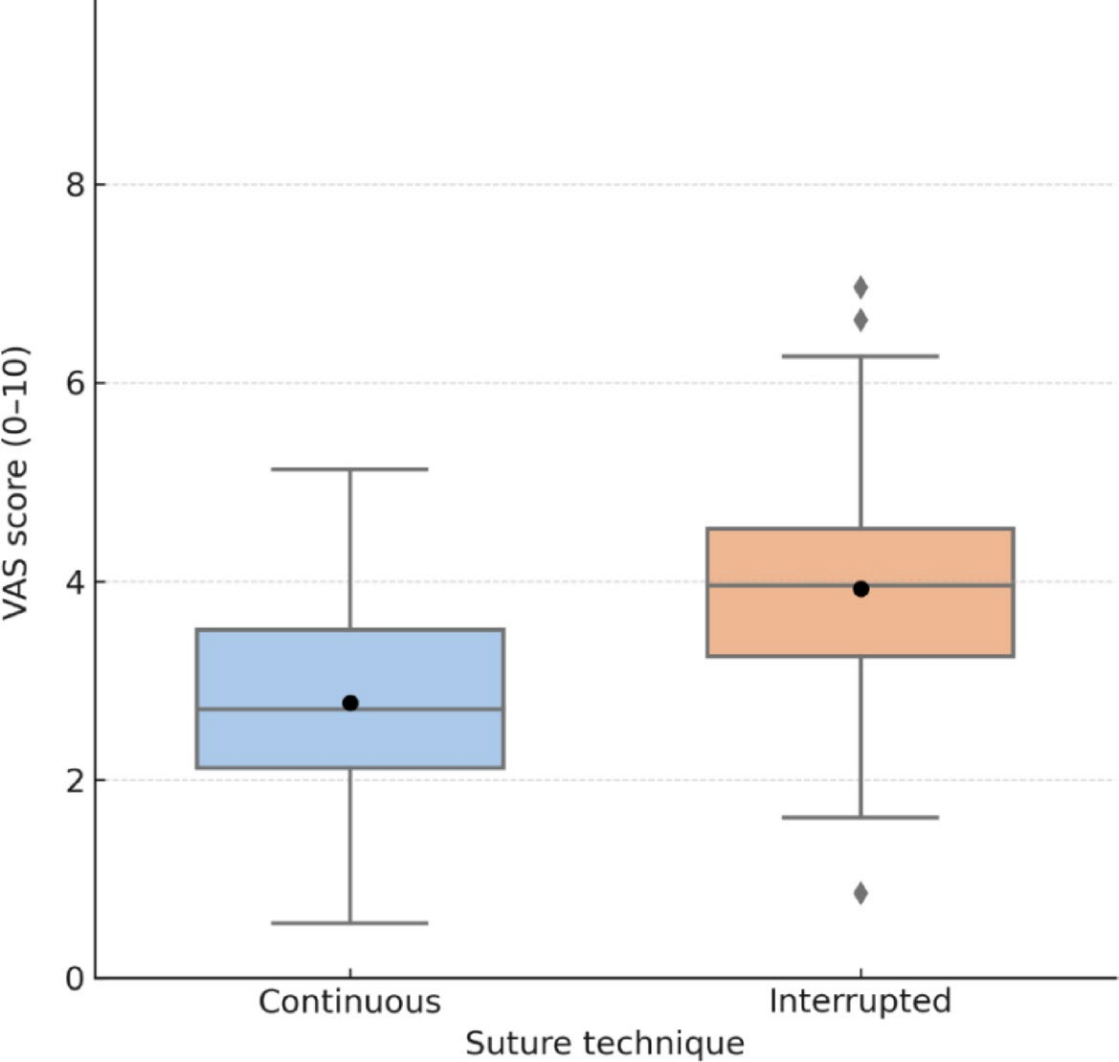
Postoperative pain at 24 h (VAS score). Boxplot comparing VAS pain scores at 24 h postoperatively between continuous and interrupted suture techniques. Horizontal lines represent medians; black dots indicate means. Whiskers reflect 1.5× IQR. Individual scores are plotted as gray dots. The continuous group showed significantly lower pain (*p* = 0.017)

Postoperative pain at 24 h was significantly lower in the continuous group (VAS 2.9 ± 2.1 vs 4.0 ± 2.1; one-sided *p* = 0.017) (Table 3; Fig. 2).

**Table 3:** Postoperative pain and wound healing

Outcome	Continuous	Interrupted	*p*-value
VAS pain score at 24 h	2.9 ± 2.1	4.0 ± 2.1	0.017
Time to complete wound healing (days)	22.3 ± 4.6	24.8 ± 5.2	0.012

VAS: Visual Analogue Scale (0–10). Pain was assessed at 24 h postoperatively. Time to wound healing was defined as the number of days from surgery to full epithelialisation without exudate, crusting, erythema, or separation of wound edges. Values are expressed as mean ± standard deviation. VAS at 24 h (primary endpoint) was analysed using a one-sided two-sample t-test (α = 0.025); time to wound healing was compared using the Mann–Whitney U test

### Secondary outcomes

Time to complete wound healing was shorter in the continuous group (22.3 ± 4.6 days) compared to the interrupted group (24.8 ± 5.2 days; *p* = 0.012). However, this variable was assessed during scheduled outpatient visits, and may have been influenced by variability in follow-up intervals.

At 6 months, the improvement in knee function (HSS score) was significantly greater in the continuous group (24.5 ± 10.5 vs 16.0 ± 12.2 points; *p* = 0.040) (Table 4).

**Table 4:** Functional and patient-reported outcomes

Outcome	Continuous	Interrupted	*p*-value
HSS score improvement at 6 months (Δ score)	24.5 ± 10.5	16.0 ± 12.2	0.040
EQ-5D index at 6 months	0.78 ± 0.12	0.75 ± 0.14	0.336
“Very satisfied” patients at 3 months (%)	88.0%	81.4%	0.379

HSS: Hospital for Special Surgery knee score. EQ-5D-3 L: EuroQol 5-Dimension 3-Level index. Patient satisfaction was measured at 3 months using a 3-point Likert scale (very satisfied, satisfied, not satisfied). Continuous variables are presented as mean ± standard deviation and were compared using Student’s t-test or the Mann–Whitney U test, as appropriate. Categorical data were analysed with the Chi-square or Fisher’s exact test, as appropriate

No significant differences were found in health-related quality of life, measured by EQ-5D index (0.78 ± 0.12 vs 0.75 ± 0.14; *p* = 0.336).

Patient satisfaction at 3 months was high in both groups: 88.0% of patients in the continuous group reported being “very satisfied” vs 81.4% in the interrupted group (*p* = 0.379).

Exploratory knee range of motion (ROM) at 6 months was high in both groups. The continuous suture group showed slightly greater flexion (mean ± SD: 113.2 ± 10.5°) compared to the interrupted group (109.5 ± 11.8°), although this difference did not reach statistical significance (Mann–Whitney *p* = 0.056).

### Complications and reinterventions

The rate of wound complications was low and comparable between groups (Table 5). Superficial or deep infection occurred in 4.2% of patients in the continuous group and 5.6% in the interrupted group (*p* = 0.410). One case of wound dehiscence (1.4%) was recorded in the continuous group; none in the interrupted group (*p* = 0.547).

**Table 5:** Wound-related complications and reinterventions

Outcome	Continuous (*n* = 72)	Interrupted (*n* = 71)	*p*-value
Superficial or deep infection (%)	3 (4.2%)	4 (5.6%)	0.410
Wound dehiscence (%)	1 (1.4%)	0 (0.0%)	0.547
Reintervention for wound issues (%)	2 (2.8%)	1 (1.4%)	0.557

Infections were defined according to the Centers for Disease Control and Prevention (CDC) criteria. Dehiscence was defined as any partial or complete separation of the wound edges. Data are presented as number of cases and corresponding percentages. Comparisons between groups were performed using Fisher’s exact test due to low event rates

Reintervention for wound-related complications was required in two patients (2.8%) in the continuous group and one (1.4%) in the interrupted group (*p* = 0.557). No cases of periprosthetic joint infection or deep revision surgery were recorded during follow-up.

## Discussion

This randomised controlled trial provides high-level evidence that continuous barbed suturing in primary total knee arthroplasty (TKA) improves surgical efficiency and early clinical outcomes without increasing complication rates. Specifically, we found significant reductions in closure time and postoperative pain, along with faster wound healing and greater functional improvement at 6 months. To our knowledge, this is the first randomised trial to demonstrate a superior functional recovery (HSS) with barbed continuous sutures compared to conventional interrupted multifilament sutures—a milestone in wound closure techniques. Consistent with this pattern, an exploratory analysis showed that 6-month knee range of motion (ROM) was high in both groups with a small, non-significant advantage for continuous suturing (113.2 ± 10.5° vs 109.5 ± 11.8°; Mann–Whitney *p* = 0.056).

Our findings align with prior reports emphasizing the clinical relevance of closure technique in joint arthroplasty. Sershon et al. [1] highlighted how minor closure-related issues such as dehiscence can significantly impact recovery and satisfaction, reinforcing the need for optimal closure methods.

The substantial reduction in closure time observed with continuous sutures (4.2 vs 6.5 min for arthrotomy) is consistent with data from hip arthroplasty by Lee et al. [2] and Sundaram et al. [3], who reported 25–40% faster closure with barbed techniques. While their focus was on the hip, the efficiency gains are generalisable to large joint surgery.

Postoperative pain scores at 24 h were significantly lower in the continuous group (VAS 2.9 vs 4.0; *p* = 0.017), in line with Smith et al. [4], whose meta-analysis reported less pain with continuous barbed sutures across orthopaedic procedures. Our trial is among the few to validate these findings in a well-powered randomised setting.

These results substantiate the international consensus of Ainslie-Garcia et al. [5], which advocated for continuous barbed sutures based on expert perceptions of efficiency, safety, and cost-effectiveness. Likewise, a national SECOT survey [6] found that most surgeons perceive barbed sutures as superior for wound approximation, reduced exposure time, and ergonomic handling—now supported by our outcome-based evidence.

Romanini et al. [7] emphasized that barbed sutures create a more hermetic closure, minimizing exudate and promoting healing. Our data mirror this conclusion: time to wound healing was shorter in the continuous group (22.3 vs 24.8 days, *p* = 0.012).

Nevertheless, the absolute difference in healing time, although statistically significant, may have limited clinical relevance. Standard TKA wounds typically heal within 14–21 days [7], and our strict definition of “complete healing” (requiring full epithelialization without erythema or crusting) may have prolonged observed times. The 2.5-day advantage thus reflects a subtle biological enhancement rather than a clearly perceptible benefit.

Moreover, healing assessments were conducted at fixed postoperative visits (days 7, 14, 30, and 90). Hence, healing time was recorded as the first documented date of epithelialization, rather than the true day of healing. This interval-censoring may slightly over- or underestimate actual healing time, although it affects both groups equally.

EFORT [8] has emphasized the theoretical advantages of barbed sutures—reduced foreign body load, less ischemia, and lower inflammation—although based on lower-level evidence. Our findings provide prospective confirmation of some of these biological benefits.

Despite expert consensus, many surgeons in Spain continue using interrupted absorbable sutures, as Sanz-Ruiz et al. [6] reported. Our data may help bridge this gap between perception and evidence, supporting a wider adoption of continuous barbed closure techniques in real-world settings.

Maniar et al. [9], in the STRIDE guidelines, recommended continuous suturing for high-tension layers like the arthrotomy to improve wound integrity. This supports our observation that the incidence of dehiscence and infection remained low and comparable between groups.

Although we did not conduct an economic analysis, prior studies suggest barbed sutures may be cost-effective due to reduced suture usage and shorter operative times [10–12]. Our results confirm the time-saving aspect, but future research is needed to assess the economic implications in different healthcare systems.

From a hospital perspective, the shorter closure time observed with continuous barbed suturing implies fewer minutes of operating-theatre occupancy and staff utilisation during wound closure. While the unit price of barbed sutures is typically higher than that of conventional absorbable sutures, the time saving and simplified handling may offset part of the material cost. Because length of stay and complication rates were similar between groups, there is no signal of increased downstream costs attributable to continuous closure in this trial. A formal, prospective micro-costing and cost-effectiveness analysis—capturing theatre time, consumables, and any unplanned visits—will be required to quantify the net budget impact across different health systems.

Continuous sutures also fit into efficiency frameworks proposed by Kashanian et al. [13], providing reproducible and time-efficient closure—especially relevant in high-volume arthroplasty centres.

Sabeh et al. [14] and Podmore et al. [15] showed that comorbidity increases complication risk. In our trial, patients with comparable Charlson Index scores had similar outcomes, reinforcing that continuous sutures are safe across varied risk profiles. Li et al. [16] and our own results found no increase in infection or reintervention rates, affirming their safety profile.

Finally, our biological rationale is supported by Luo et al. [17], who reported that running sutures reduce periwound inflammation and exudate. Masuda et al. [18] further showed better early cosmesis and satisfaction with barbed skin closure in TKA—reinforcing our findings of reduced pain and faster healing. In this context, the small, non-significant ROM advantage observed with continuous suturing may reflect a trajectory of earlier comfortable mobilisation that ultimately converges by 6 months; prospective ROM collection at predefined time points will clarify whether earlier differences translate into durable motion gains.

Importantly, our study demonstrated a significantly greater improvement in functional recovery (HSS score) in the continuous group (24.5 vs 16.0 points). This difference—exceeding 8 points—approaches or surpasses the lower range of reported MCID values for HSS (9–18 points). While modest early differences in pain and healing may not fully explain this, we hypothesize that early comfort may facilitate better ambulation, faster rehabilitation progression, and more favourable gait mechanics. Additionally, lower inflammation and stiffness due to uniform tension distribution may contribute to long-term joint mobility.

A key strength of our study lies in its double-blind randomised design, uniform surgical technique, and validated outcome measures. All procedures were performed by experienced surgeons using the same implant, reducing variability.

However, certain limitations must be acknowledged. This was a single-centre trial, which may limit generalisability. The 6-month follow-up suffices for early outcomes but not for long-term endpoints such as late stiffness, satisfaction durability, or implant survival. Moreover, while the study was powered for pain and closure time, it was underpowered to detect rare adverse events such as deep infection or late revision. Thus, non-significant differences in rare outcomes should not be interpreted as proof of equivalence. Additionally, ROM was obtained from physiotherapy records and analysed post hoc as an exploratory endpoint; incomplete availability and measurement variability may have limited power to detect small between-group differences. Hospital costs and resource use were not prospectively captured; consequently, economic interpretations are qualitative and hypothesis-generating.

Closure time is also operator-dependent. Although both surgeons were proficient in both techniques, individual preferences may have influenced the results. Future multicentre studies including stratified surgeon-level analyses are warranted.

In summary, continuous barbed sutures demonstrated clear advantages over interrupted multifilament sutures in primary TKA, including faster closure, reduced early pain, and enhanced short-term functional recovery—without increasing complication or reintervention rates. Importantly, this is the first randomised trial to demonstrate a significantly greater functional improvement (HSS) with continuous barbed closure, marking a true milestone in arthrotomy management. Exploratory ROM findings were directionally consistent with this pattern but did not reach statistical significance.

These findings support the adoption of continuous closure techniques, particularly in high-volume centres and enhanced recovery programmes. Nonetheless, multicentre studies with extended follow-up and formal cost-effectiveness analyses are warranted to confirm the generalizability and long-term value of this approach. Subgroup analyses based on patient-specific factors (e.g., obesity, diabetes) may also guide more personalized wound closure strategies.

## Conclusion

Continuous barbed suturing in primary TKA significantly reduces closure time and early postoperative pain, shortens wound healing time, and improves early functional outcomes without increasing complication rates. This is the first randomised trial to demonstrate superior HSS improvement with continuous barbed versus interrupted multifilament sutures. These findings support the routine use of barbed sutures in TKA closure, particularly in fast-track protocols. Although hospital costs were not prospectively collected, the observed efficiency gains suggest potential hospital-level economic benefits that warrant prospective micro-costing and cost-effectiveness evaluation.

## Data Availability

The datasets generated and analysed during this study are not publicly available due to institutional restrictions and patient confidentiality, but may be made available by the corresponding author upon reasonable request.
